# Factors Associated with Prevalence, Awareness, Treatment and Control of Hypertension among Adults in Southern China: A Community-Based, Cross-Sectional Survey

**DOI:** 10.1371/journal.pone.0062469

**Published:** 2013-05-09

**Authors:** Hao Wang, Xinwei Zhang, Jie Zhang, Qingfang He, Ruying Hu, Lixin Wang, Danting Su, Yuanyuan Xiao, Jin Pan, Zhen Ye

**Affiliations:** 1 Department of Chronic Non-Communicable Diseases Control and Prevention, Zhejiang Provincial Center for Disease Control and Prevention, Hangzhou, Zhejiang, China; 2 General office, Health Bureau of Zhejiang Province, Hangzhou, Zhejiang, China; University of Chieti, Italy

## Abstract

The aim of this study was to investigate factors associated with prevalence, awareness, treatment and control of hypertension in southern China. A cross-sectional, population-based survey was conducted in 180 villages across 15 counties in southern China from July to November 2010. Totally, 17437 persons completed all of the questionnaires, measurement examination and blood specimen collection. Adjusted rate of hypertension was 22.59% (95%CI: 22.52%–22.66%), for men 23.36% (95%CI: 23.25%–23.47%) and for women 21.77% (95%CI: 21.68%–21.86%). Multivariate logistic regression showed that old age, education attainment, alcohol use, diabetes, obesity, high TC and high TG were associated with hypertension. Among the hypertensive individuals, 54.33% were aware of their hypertension, and 46.34% were currently taking antihypertensive medication, but only 18.26% had their blood pressure controlled. Among all the hypertensive subjects, awareness was more common in those who were female, living in the urban, old age, low education attainment, diabetes, overweight, obese, Low HDL-C. Among the subjects aware of their diagnosis of hypertension, medication treatment was more common in those living in the urban, old age, nonsmoker and diabetes. Among the individuals who receiving medication treatment, controlled hypertension were less common in those living in the urban, young age, low education attainment, overweight and obese. Despite high rate of hypertension, awareness, treatment and control of hypertension still need to be strengthened.

## Introduction

With the rapid change of lifestyle and dietary pattern, cardiovascular disease (CVD) has become the leading cause of death in China [1]. Hypertension is the most important risk factor of CVD worldwide, contributing to one half of the coronary heart disease and approximately two thirds of the cerebrovascular disease burdens [2]. A national nutrition and health survey conducted in 2002 showed that the prevalence of hypertension among adults aged ≥18 years was 18%, which indicated there were 153 million people with hypertension in China. Of those, less than one quarter was aware of their condition. Little improvement had been made in either the prevalence or awareness of the condition, compared with the survey in 1991 [3].

Blood pressure (BP) and prevalence of hypertension are greater for northern than southern Chinese [3], [4]. Several studies of prevalence, awareness, treatment and control have been conducted in China, but the majority of those were carried out in the northern China [5]–[7]. Few studies have been implemented in the southern China. Zhejiang province, one of the most developed and wealthiest provinces, lies in the south of China and has a population of 56 million. The result from Zhejiang provincial nutrition and health survey in 2002 showed that the prevalence of hypertension among adults was 19.8%. The rates of awareness, treatment and control among hypertensives were 37.13%, 29.61% and 10.15%, respectively [8]. But the up-to-date prevalence, the rates of awareness, treatment and control were unknown. Establishing factors associated with awareness and management is an essential starting point in preventing increasing burden of morbidity and mortality from hypertension-related cardiovascular diseases [9]. However, information on factors associated with awareness treatment and control in adults in southern china is very scarce. To explore the prevalence, awareness, treatment and control of hypertension in the southern China, 17437 subjects from Zhejiang province were surveyed in 2010.

## Methods

### Ethics statement

This study was approved by the ethnics committee of Zhejiang Provincial Center for Disease Control and Prevention. Written informed consent was obtained from each participant. The ethics committee approved this procedure.

### Subjects and study design

This study was a community-based, cross-sectional survey conducted in Zhejiang province. We used multistage stratified random cluster sampling to select the study household. In the first stage, all of the 91 counties were divided into Type 1 urban districts, Type 2 urban districts, Type 1 rural counties, Type 2 rural counties and Type 3 rural counties, based on economic levels. From each of these five groups we systematically selected three counties. Totally 15 counties were chosen at the first stage. In the second stage, four streets were selected randomly within each chosen county. In the third stage, three villages were selected randomly within each chosen street. In the fourth stage, all the households in the chosen villages were divided into different clusters based on the adjacent geographic location. Every cluster was composed of forty households. One cluster was selected randomly from a number of clusters in one village. Subjects were excluded if they meet any of the following items during the survey period: stroke, dementia, schizophrenia, ill in bed, deaf and dumb, <18 years old, less than six months of living in the local. All subjects aged ≥18 years in the chosen households and fail to meet exclusion criteria were included in the survey.

County-level disease control and prevention centers and community health service centers were responsible for mobilizing and implementing the field work. The trained investigators visited the households and gave written informed consents to them prior to the survey. A central survey site was set up in each residential committee or village and the participants were required to be interviewed and receive the health examination on-site. All interviews and examinations were conducted following standardized protocols. A standard questionnaire was administered by trained investigator to obtain information on demographic characteristics, family history of chronic diseases, lifestyle risk factors, and condition about diagnosis and treatment of hypertension, diabetes and dyslipidemia. Height, weight were measured after participants taking off their shoes, hats, coats, and sweaters.

After at least 10 hours of overnight fasting, venous blood specimen was collected in vacuum tubes, for the measurement of fasting blood glucose (FBG) and blood-lipid. Participants with no history of diabetes and 5.0 mmol/L≤FBG<7.0 mmol/L, were appointed for oral glucose tolerance test (OGTT) in other days. Standard 75 g glucose solutions were given to participants to drink. Blood specimens were collected after 2 hours to measure glucose concentration.

The standardized mercury sphygmomanometers were used for the measurement in the present study. Participants were advised to avoid alcohol, cigarette smoking, coffee/tea and exercise for at least 30 min before their BP measurement. BP was measured after the subject had rested for at least 5 minutes by a trained and certified observer. Two consecutive readings of BP were taken on the right arm according to 1999 World Health Organization/International Society of Hypertension guidelines on hypertension [10]. Systolic blood pressure (SBP) was defined as the average of two SBP readings. Diastolic blood pressure (DBP) was defined as the average of two DBP readings. We defined hypertension as SBP≥140 mmHg, DBP≥90 mmHg, and/or use of antihypertensive medications within the 2 weeks before the interview. Awareness of hypertension was defined as self–report of any prior diagnosis of hypertension by a health-care professional among the population defined as having hypertension. Treatment of hypertension was defined as use of a prescription medication for management of high BP before the two weeks. Control of hypertension was defined as pharmacological treatment of hypertension associated with an average SBP<140 mmHg and an average DBP<90 mmHg. Body mass index (BMI) was calculated as the ratio of the weight to the square of the height(kg/m^2^).Overweight was defined as 23≤BMI<25; Obesity was defined as BMI≥25.0 [11]. Diabetes mellitus was diagnosed according to WHO criteria in subjects with FBG≥7.0 mmol/L (126 mg/dL) or 2 h glucose level ≥11.1 mmol/L (200 mg/dL) or who had previously been diagnosed with diabetes by a physician. Individuals who smoked 1 cigarette per day for over 6 months were defined as smokers, and those who consumed at least 1 time every one week were considered as alcohol drinkers. High total cholesterol (TC) was defined as a TC concentration ≥6.22 mmol/L (240 mg/dL); Low high density lipoprotein cholesterol (HDL-C) was defined as a HDL-C concentration <1.04 mmol/L (40 mg/dL); High Triglycerides (TG) was defined as a TG concentration ≥2.26 mmol/L (200 mg/dL). Plasma glucose was measured with the glucose oxidase method. Lipids were measured using Beckman autoanalyzer at the biochemistry laboratory of Zhejiang provincial centre for disease control and prevention.

All interviewers and staff members completed a training program that familiarized them with both the aims of the study and the specific tools and methods used. At the specific sessions, interviewers were given detailed instructions concerning the administration of the study questionnaire. Clinic staff members were taught to measure BP and acquire anthropometric measurements.

### Statistical analysis

Continuous variables were given as the mean±s.d. The prevalence of hypertension was given as percent and 95% confidence intervals (CI). Age- sex-and -region adjustment were performed using the direct method and Standards came from 2010 Zhejiang provincial population census. Categorical variables between groups were performed using chi square. Multivariate logistic regression was used to assess factors associated with hypertension. We first determine which factors were associated with hypertension in univariate analyses (*P*<0.05).Variables significant in the univariate analyses were entered in a multivariate logistic regression model that was performed by a backward stepwise logistic regression. All analyses were performed with SPSS statistical software, version 13.0 (Statistical Package for the Social Sciences, SPSS Inc, Chicago, Illinois). All statistical tests were two tailed, and *P*-values<0.05 were considered statistically significant.

## Results

A total of 19113 subjects were selected and invited to participate in the study. 583 refused to participate in the survey. 913 subjects were not at home during the period of survey. A total of 180 failed to collect blood specimen. Virtually, 17437 subjects (8169 men and 9268 women) (table 1) completed all of the questionnaires, measurement examination and blood specimen collection. The overall response rate was 91.23%: 87.96% for the urban regions and 93.51% for the rural regions.

**Table 1:** Characteristics of the subjects surveyed in southern China in 2010 (N = 17437).

Variables	Total (N = 17437)		Urban (N = 6912)		Rural (N = 10525)	
	n	%	n	%	n	%
Age groups						
18–44	6552	37.58	2310	33.42	4242	40.30
45–59	6293	36.09	2515	36.39	3778	35.90
≥60	4592	26.33	2087	30.19	2505	23.80
Gender						
Female	9268	53.15	3741	54.12	5527	52.51
Male	8169	46.85	3171	45.88	4998	47.49
Education						
College or higher	836	4.79	388	5.61	448	4.26
Middle	7565	43.38	2902	41.98	4663	44.30
Primary	5727	32.84	2206	31.92	3521	33.45
Illiterate	3309	18.98	1416	20.49	1893	17.99
Income per capita						
≥20000 Yuan	7284	41.77	3567	51.61	3717	35.32
10000–19999Yuan	4675	26.81	1600	23.15	3075	29.22
<10000 Yuan	5478	31.42	1745	25.25	3733	35.47
Type of smoke use						
Nonsmoker	12206	70.00	4944	71.53	7262	69.00
Smoker	5231	30.00	1968	28.47	3263	31.00
Type of alcohol use						
Nondrinker	12194	69.93	4731	68.45	7463	70.91
Drinker	5243	30.07	2181	31.55	3062	29.09
Diabetes						
No	15908	91.23	6129	88.67	9779	92.91
Yes	1529	8.77	783	11.33	746	7.09
BMI						
<23	8864	50.83	3441	49.78	5423	51.52
23–24.99	3837	22.00	1548	22.40	2289	21.75
≥25	4736	27.16	1923	27.82	2813	26.73
TG						
<2.26	15166	86.98	6122	88.57	9044	85.93
≥2.26	2271	13.02	790	11.43	1481	14.07
TC						
<6.22	16995	97.47	6700	96.93	10295	97.81
≥6.22	442	2.53	212	3.07	230	2.19
HDL-C						
≥1.04	10203	58.51	4116	59.55	6087	57.83
<1.04	7234	41.49	2796	40.45	4438	42.17

BMI: Body mass index. TG: Triglycerides. TC: Total cholesterol. HDL-C: high density lipoprotein cholesterol.

Of the 17437 respondents (mean 49.30±15.10 years old), 5227 subjects (mean 58.09±12.51 years old) were diagnosed as hypertension. The crude rate of hypertension was 29.98% (95%CI: 29.30%–30.66%). The crude rates of men and women were 31.22% (95%CI: 30.21%–32.22%) and 28.88% (95%CI: 27.96%–29.81%) (*χ^2^* = 11.24 *P*<0.01). Adjusted rate of hypertension was 22.59% (95%CI: 22.52%–22.66%). Adjusted rate of men and women were 23.36% (95%CI: 23.25%–23.47%) and 21.77% (95%CI: 21.68%–21.86%).

**Figure 1:**
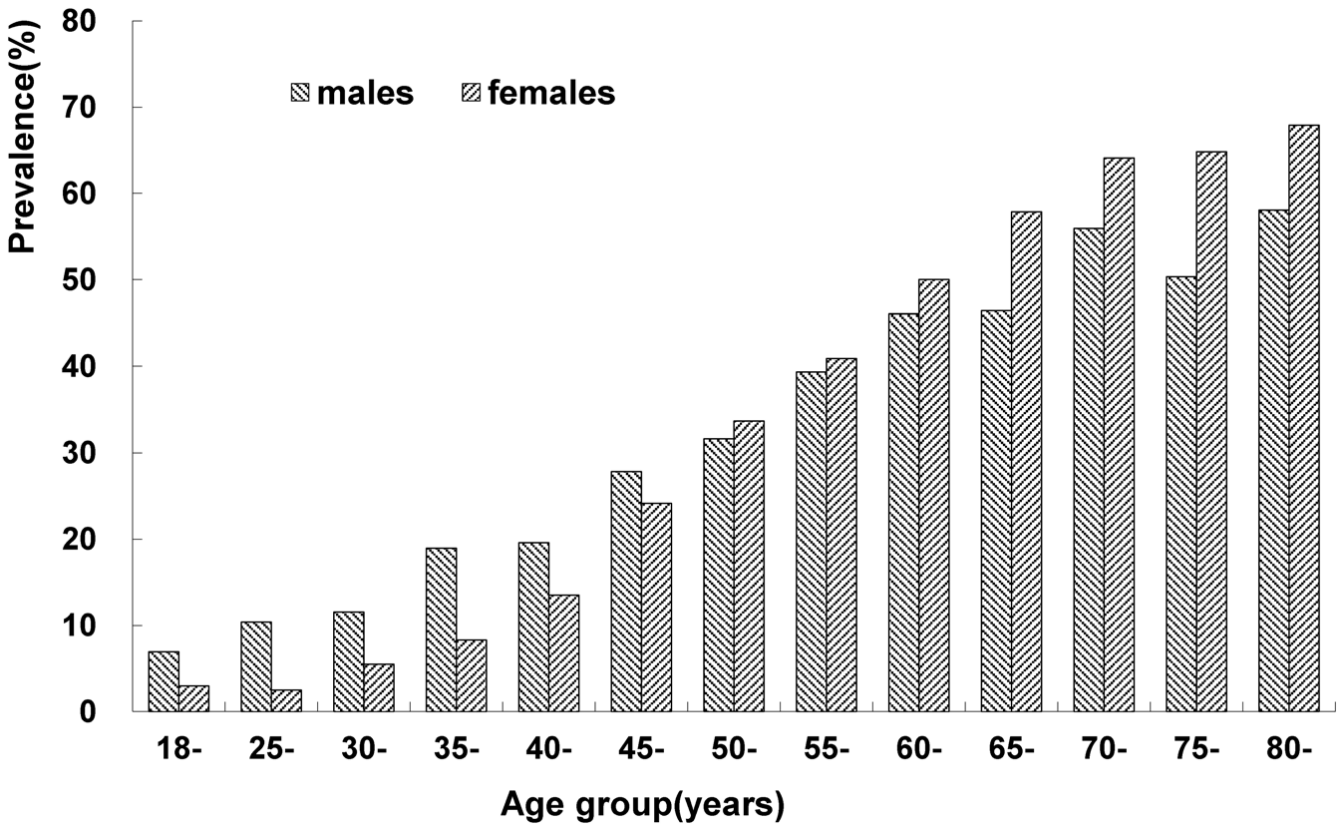
Prevalence of hypertension among adults of different age groups in southern China in 2010 (N = 17437).

Figure 1 showed that the prevalence of hypertension increased with increase in age, and this linear trend was statistically significant (*χ^2^* for linear trend = 2464.34 *P*<0.001). The prevalence of hypertension among residents aged 18–24, 25–29, 30–34, 35–39, 40–44, 45–49, 50–54, 55–59, 60–64, 65–69, 70–74, 75–79, ≥80 years were 4.77%, 5.67%, 8.26%, 13.15%, 16.26%, 25.80%, 32.73%, 40.15%, 48.06%, 51.94%, 59.54%, 56.82%, 63.31%, respectively.

### Sociodemographic characteristics and factors

Table 2 showed that old age is associated with higher prevalence of hypertension (OR = 3.37 [age 45–59] and 8.10 [age ≥60] vs age 18–44). Lower education is associated with higher prevalence of hypertension(OR = 1.22 [education: primary] and 1.43 [education: illiterate] vs college or higher. Drinker has a higher risk of having hypertension than nondrinker (OR = 1.20). People with higher BMI are more likely to be hypertensive patients (OR = 1.70 [BMI = 23–24.99] and 3.27 [BMI≥25] vs BMI<23). People who had diabetes, high TG and high TC have higher risk of having hypertension (OR = 2.11, 1.59, and 1.38, respectively).

**Table 2:** Crude and adjusted odd ratio of factors associated with hypertension in southern China in 2010 (N = 17437).

Variable	Total (N = 17437)		Urban(N = 6912)		Rural (N = 10525)	
	COR(95%CI)	AOR(95%CI)	COR(95%CI)	AOR(95%CI)	COR(95%CI)	AOR(95%CI)
Gender(ref: Female)	1.12(1.05–1.19)		1.18(1.07–1.31)		1.08(0.99–1.18)	
Age groups(ref: 18–44 years)						
45–59	4.22(3.84–4.64)	3.37(3.04–3.74)	4.01(3.46–4.65)	3.17(2.70–3.73)	4.31(3.81–4.88)	3.44(3.00–3.94)
≥60	9.53(8.64–10.51)	8.10(7.23–9.07)	8.92(7.68–10.37)	7.24(6.11–8.59)	9.66(8.49–10.99)	8.49(7.29–9.89)
Education						
(ref: College or higher)						
Middle	1.77(1.44–2.18)	1.09(0.87–1.37)	1.69(1.28–2.22)	1.15(0.84–1.57)	2.11(1.52–2.91)	1.13(0.80–1.60)
Primary	3.48(2.83–4.28)	1.22(0.97–1.55)	2.94(2.24–3.87)	1.27(0.92–1.75)	4.55(3.30–6.28)	1.33(0.94–1.89)
Illiterate	5.24(4.25–6.47)	1.43(1.12–1.84)	4.31(3.26–5.70)	1.51(1.09–2.10)	6.88(4.96–9.54)	1.53(1.07–2.19)
Income per capita						
(ref: ≥20000 Yuan)						
10000–19999Yuan	0.94(0.86–1.02)		0.97(0.86–1.10)	0.93(0.80–1.06)	1.05(0.94–1.17)	
<10000 Yuan	1.09(1.01–1.17)		0.84(0.74–0.95)	0.79(0.68–0.90)	1.44(1.30–1.60)	
Cigarette use(ref: Nonsmoker)	1.02(0.95–1.09)		0.98(0.88–1.10)		1.07(0.97–1.17)	
Alcohol use(ref: Nondrinker)	1.34(1.25–1.43)	1.20(1.10–1.31)	1.31(1.18–1.45)	1.24(1.10–1.39)	1.34(1.22–1.47)	1.19(1.07–1.32)
Diabetes(ref: No)	3.76(3.38–4.19)	2.11(1.87–2.37)	3.90(3.34–4.56)	2.33(1.97–2.77)	3.41(2.94–3.97)	1.82(1.54–2.14)
BMI(ref: <23)						
23–24.99	1.93(1.77–2.10)	1.70(1.55–1.87)	1.97(1.73–2.24)	1.74(1.51–2.01)	1.90(1.69–2.12)	1.67(1.48–1.89)
≥25	3.45(3.19–3.73)	3.27(3.00–3.56)	3.58(3.18–4.04)	3.23(2.83–3.69)	3.36(3.04–3.73)	3.29(2.94–3.69)
High TG(ref: <2.26)	2.11(1.93–2.30)	1.59(1.44–1.77)	2.24(1.93–2.60)	1.66(1.40–1.97)	2.12(1.89–2.38)	1.64(1.44–1.86)
High TC(ref: <6.22)	2.29(1.89–2.76)	1.38(1.12–1.70)	2.45(1.86–3.23)	1.59(1.17–2.17)	2.04(1.57–2.66)	
Low HDL-C(ref: ≥1.04)	1.10(1.03–1.18)		1.17(1.06–1.29)		1.07(0.98–1.16)	

COR: Crude odds ratio. AOR: Adjusted odds ratios. CI: confidence intervals. BMI: Body mass index. TG: Triglycerides. TC: Total cholesterol. HDL-C: high density lipoprotein cholesterol.

### Awareness, treatment and control of hypertension

Just as showed in table 3, of the 5227 hypertensive individuals, 2840 (men 1319 and women 1521) had been aware of their condition prior to the survey. The overall awareness rate of hypertension was 54.33%. There was significant difference between men and women of awareness of hypertension (51.73% vs 56.82%, *P*<0.001). 2422 hypertensive individuals had used prescription medication for management of high BP before the two weeks. The overall treatment rate of hypertension was 46.34%.Women had a higher treatment rate than men (49.05% vs 43.49% *P*<0.001). Only 18.26% had their BP under control. There was no significant association between men and women (17.92% vs 18.57% *P*>0.05). Table 4 showed that 73.34% of the individuals with hypertension among 18–44 age group were unaware of having hypertension, making up 21.32% of all those unaware of hypertension.

**Table 3:** Percentage (%) of awareness, treatment and control of hypertension among hypertensive adults (N = 5227) in Southern China in 2010.

		Awareness			Treatment			Control		
	N^a^	n^b^(%)	(95%CI)	*P*	n^c^(%)	(95%CI)	*P*	n^d^(%)	(95%CI)	*P*
Total	5227	2840(54.33)	52.98–55.68		2422(46.34)	44.98–47.69		954(18.26)	17.20–19.30	
Men	2550	1319(51.73)	49.78–53.67	<0.001	1109(43.49)	41.56–45.42	<0.001	457(17.92)	16.43–19.41	>0.05
Women	2677	1521(56.82)	54.94–58.69		1313(49.05)	47.15–50.94		497(18.57)	17.09–20.04	
Urban										
Total	2386	1389(58.21)	56.23–60.19		1223(51.26)	49.25–53.26		455(19.07)	17.49–20.65	
Men	1160	653(56.29)	53.43–59.15	>0.05	570(49.14)	46.26–52.02	<0.05	219(18.88)	16.62–21.13	>0.05
Women	1226	736(60.03)	57.29–62.78		653(53.26)	50.47–56.06		236(19.25)	17.04–21.46	
Rural										
Total	2841	1451(51.07)	49.23–52.91		1199(42.20)	40.39–44.02		499(17.56)	16.16–18.96	
Men	1390	666(47.91)	45.28–50.54	<0.01	539(38.78)	36.21–41.34	<0.001	238(17.12)	15.14–19.11	>0.05
Women	1451	785(54.10)	51.53–56.67		660(45.49)	42.92–48.05		261(17.99)	16.01–19.97	

a Number of hypertensives.

b Number of hypertensives who were aware of their diagnosis of hypertension.

C Number of hypertensives receiving antihypertensive medication.

d Number of hypertensives whose blood level under control.

**Table 4:** Characteristics of the subjects with hypertension and those aware of hypertension and those receiving antihypertensive medication.

Variables	Awareness (N = 5227)^a^		Treatment(N = 2840)^b^		Control (N = 2422)^c^	
	Unaware	Aware	Untreated	Treated	Uncontrolled	Controlled
Region						
Rural	1390	1451(51.07)†	252	1199(82.63)†	700	499(41.62)*
Urban	997	1389(58.21)	166	1223(88.05)	768	455(37.20)
Age(in years)						
18–44	509	185(26.66)§	48	137(74.05)§	95	42(30.66)§
45–59	1029	1069(50.95)	192	877(82.04)	511	366(41.73)
≥60	849	1586(65.13)	178	1408(88.78)	862	546(38.78)
Gender						
Female	1156	1521(56.82)†	208	1313(86.32)	816	497(37.85)
Male	1231	1319(51.73)	210	1109(84.08)	652	457(41.21)
Education						
College or higher	71	41(36.61)§	8	33(80.49)	16	17(51.51)§
Middle	815	813(49.94)	118	695(85.49)	384	311(44.75)
Primary	877	1128(56.26)	164	964(85.46)	571	393(40.77)
Illiterate	624	858(57.89)	128	730(85.08)	497	233(31.92)
Income per capita						
≥20000 Yuan	969	1201(55.35)	159	1042(86.76)	633	409(39.25)
10000–19999Yuan	604	725(54.55)	112	613(84.55)	352	261(42.58)
<10000 Yuan	814	914(52.89)	147	767(83.92)	483	284(37.03)
Type of smoke use						
Nonsmoker	1635	2009(55.13)	270	1739(86.56)*	1085	654(37.61)**
Smoker	752	831(52.50)	148	683(82.19)	383	300(43.92)
Type of alcohol use						
Nondrinker	1514	1914(55.83)**	261	1653(86.36)*	989	664(40.17)
Drinker	873	926(51.47)	157	769(83.05)	479	290(37.71)
Diabetes						
No	2116	2217(51.47)†	350	1867(84.21)**	1132	735(39.37)
Yes	271	623(69.69)	68	555(89.09)	336	219(39.46)
BMI						
<23	885	893(50.22)§	129	764(85.55)	424	340(44.50)§
23–24.99	551	701(55.99)	109	592(84.55)	362	230(38.85)
≥25	951	1246(56.71)	180	1066(85.55)	682	384(36.02)
TG						
<2.26	1927	2284(54.24)	322	1962(85.90)	1180	782(39.86)
≥2.26	460	556(54.72)	96	460(82.73)	288	172(37.39)
TC						
<6.22	2298	2713(54.14)	401	2312(85.22)	1393	919(39.75)
≥6.22	89	127(58.80)	17	110(86.61)	75	35(31.82)
HDL-C						
≥1.04	1423	1550(52.14)†	239	1311(84.58)	820	491(37.45)*
<1.04	964	1290(57.23)	179	1111(86.12)	648	463(41.67)

a Among subjects with hypertension.

b Among subjects aware of their diagnosis of hypertension.

c Among subjects receiving antihypertensive medication.

* *P*<0.05.

** *P*<0.01.

† *P*<0.001.

§ *P*-trend<0.001.

BMI: Body mass index. TG: Triglycerides. TC: Total cholesterol. HDL-C: high density lipoprotein cholesterol.

Table 5 showed that among those with hypertension, old age (OR = 2.83 [age 45–59] and 5.37[age≥60] vs age 18–44), living in the urban (OR = 1.26), low education attainment (OR = 1.72 [education: middle] and 1.69 [education: primary] vs education: college or higher), diabetic hypertensive (OR = 1.86), overweight (OR = 1.28), obese (OR = 1.44), low HDL-C (OR = 1.25) were associated with higher awareness of hypertension. In contrast, male was associated with lower awareness (OR = 0.84). Among those aware of hypertension, old age (OR = 1.59 [age 45–59] and 2.70 [age≥60] vs age 18–44), living in the urban (OR = 1.50), diabetic hypertensive (OR = 1.36) were associated with higher treatment of hypertension. In contrast, smoker was associated with lower treatment (OR = 0.71). Among participants receiving treatment for their hypertension, old age (OR = 1.81 [age 45–59] and 1.63[age≥60] vs age 18–44) was associated with higher control of hypertension. In contrast, living in the urban (OR = 0.81), low education attainment (OR = 0.41[education: illiterate] vs education: college or higher), overweight (OR = 0.76), obese (OR = 0.67), were associated with lower control.

**Table 5:** Crude and adjusted odd ratio of awareness, treatment, control of hypertension among adults in southern China in2010.

Variable	Awareness(N = 5227)^a^^,^ *		Treatment(N = 2840)^b^^, ^ †		Control(N = 2422)^c^^, ^ §	
	COR(95%CI)	AOR(95%CI)	COR(95%CI)	AOR(95%CI)	COR(95%CI)	AOR(95%CI)
Gender(ref: Female)	0.81(0.73–0.91)	0.84(0.75–0.95)	0.84(0.68–1.03)		1.15(0.98–1.36)	
Region(ref: Rural)	1.34(1.20–1.49)	1.26(1.12–1.41)	1.55(1.25–1.91)	1.50(1.21–1.86)	0.83(0.71–0.98)	0.81(0.68–0.96)
Age groups(ref: 18–44 years)						
45–59	2.86(2.37–3.45)	2.83(2.32–3.45)	1.60(1.11–2.30)	1.59(1.10–2.30)	1.62(1.10–2.39)	1.81(1.22–2.68)
≥60	5.14(4.26–6.20)	5.37(4.38–6.59)	2.77(1.93–3.99)	2.70(1.87–3.90)	1.43(0.98–2.09)	1.63(1.11–2.41)
Education(ref: College or higher)						
Middle	1.73(1.16–2.57)	1.72(1.12–2.64)	1.43(0.64–3.17)		0.76(0.38–1.53)	0.74(0.37–1.50)
Primary	2.23(1.50–3.30)	1.69(1.08–2.57)	1.42(0.65–3.14)		0.65(0.32–1.30)	0.59(0.29–1.19)
Illiterate	2.38(1.60–3.55)	1.44(0.93–2.24)	1.38(0.62–3.06)		0.44(0.22–0.89)	0.41(0.20–0.83)
Income per capita						
(ref: ≥20000 Yuan)						
10000–19999Yuan	0.97(0.84–1.11)		0.84(0.6–1.08)		1.15(0.94–1.41)	
<10000 Yuan	0.91(0.80–1.03)		0.80(0.62–1.02)		0.91(0.75–1.10)	
Cigarette use(ref: Nonsmoker)	0.90(0.80–1.01)		0.72(0.58–0.89)	0.71(0.57–0.89)	1.30(1.09–1.56)	
Alcohol use(ref: Nondrinker)	0.84(0.75–0.94)		0.77(0.62–0.96)		0.90(0.76–1.08)	
Diabetes(ref: No)	2.19(1.88–2.56)	1.86(1.58–2.18)	1.53(1.16–2.02)	1.36(1.03–1.81)	1.00(0.83–1.22)	
BMI(ref: <23)						
23–24.99	1.26(1.09–1.46)	1.28(1.10–1.49)	0.92(0.70–1.21)		0.79(0.64–0.99)	0.76(0.61–0.95)
≥25	1.30(1.15–1.47)	1.44(1.25–1.64)	1.00(0.78–1.28)		0.70(0.58–0.85)	0.67(0.55–0.82)
High TG(ref: <2.26)	1.02(0.89–1.17)		0.79(0.61–1.01)		0.90(0.73–1.11)	
High TC(ref: <6.22)	1.21(0.92–1.59)		1.12(0.67–1.89)		0.71(0.47–1.07)	
Low HDL-C(ref: ≥1.04)	1.23(1.10–1.37)	1.25(1.11–1.41)	1.13(0.92–1.40)		1.19(1.01–1.41)	

a Among subjects with hypertension.

* Adjusted for gender, region, age, education attainment, diabetes, BMI, HDL-C.

b Among subjects aware of hypertension.

† Adjusted for region, age, cigarette use, diabetes.

c Among subjects receiving antihypertensive medication.

§ Adjusted for region, age, education attainment, BMI.

COR: Crude odds ratio. AOR: Adjusted odds ratios. CI: confidence intervals. BMI: Body mass index. TG: Triglycerides. TC: Total cholesterol. HDL-C: high density lipoprotein cholesterol.

## Discussion

Hypertension is a major independent risk factor for cardiovascular disease and stroke. It has been estimated that more than one quarter of the world adult population had hypertension in the year 2000, and that this would increase to 29% by the year 2025 [12]. Our study indicates that adjusted prevalence of hypertension among adults was 22.59%. Accordingly, there are 10.2 million hypertension individuals in Zhejiang. Compared with the prevalence (19.8%) in 2002 [8], the prevalence of hypertension in 2010 has increased by 14%. One explanation for this increase may be the aging of the population. Another explanation may be changes in lifestyle and diet. The prevalence of hypertension in the present study is lower than those in northern provinces china, such as Beijing(35.5%) [5], Liaoning(28.7%) [6]. Due to different dietary habits, People living in the northern China usually take in more salt than those living in the south [4], [13]. Resident of Zhejiang usually better like low-salt and low-fat diet.

The results of this study indicate that the prevalence of hypertension increased with increase in age. This finding is consistent with other studies [14]–[17]. Nearly one half of the elders aged 60–64 years have hypertension. This suggests that the elders are at high risk of hypertension. Therefore, the elders should be key crowd of hypertension prevention and control. Health education and intervention measures should be implemented to prevent and control hypertension among the elders. This study also demonstrates that, for the residents less than 50 years old, men had a higher rate of hypertension than women, but for those at least 50 years the reverse was true, similar with the result of previous study [6]. Women usually enter the menopause, accompanied by a dramatic rise in the prevalence of hypertension, at the age of 50 years old, which suggests losing a protective role of endogenous estradiol on BP [18].

Increasing the awareness, treatment and control of hypertension will reduce morbidity and mortality of cardiovascular diseases [19]. Compared with the survey in 2002, the awareness treatment and control of hypertension among adults in Zhejiang province increased from 37.13% to 54.33%, from 29.61% to 46.34%, from 10.15% to 18.26%, respectively. There are several reasons resulting in these increases. First, governments implemented Farmer Health Examination Project (FHEP) in 2006, community health service centers provide all of the local farmers with free health examination once in two years. More and more hypertensive patients are found earlier through FHEP. Second, governments carried out Basic Health Services (BHS), community doctors establish health record for every resident, and follow up regularly hypertensive patients, and guide them antihypertensive drug use, and evaluate the effect of management. Third, governments implement essential drug system, reducing drug circulation link, lowering the price of antihypertensive drugs. More and more hypertensive patients can afford to eat medicine. The study [5] surveyed in Beijing showed that the hypertension awareness treatment and control rates among adults aged 18–79 years were 42.5%, 35.9% and 11.8%, which are lower than the findings in this study. Although hypertension awareness, treatment and control have improved in recent years in Zhejiang province, they compared with those of developed countries, there are major differences. The National Health and Nutrition Examination Surveys (NHANES) among American population in 2007–2008 indicated that hypertension awareness treatment and control rates were 80.6%, 73.7% and 30.3% [20], which were much higher than the findings in this study. Our results showed hypertension awareness and treatment in women were higher than in men which are commonly observed results either in developed countries [21], [22] or in the developing countries [23]. Compared to women, men visit the doctor less often, have shorter consultations, and tend to see their physician later in the course of their illness. Vigna-Taglianti F *et al* also found that gender specific effects of comprehensive social influence programmes among girls and boys because of developmental and personality factors [24]. These explanations may be considered as the reasons why women have higher hypertension awareness and treatment.

Most guidelines advocate screening for blood pressure from a relatively young age as well as increased screening frequency among those in high-risk groups [25], [26]. However, In our study, approximately three quarter of the individuals with hypertension among 18–44 age group were unaware of having hypertension, making up 21.32% of all those unaware of hypertension. Thus, screening strategies need to be designed to appeal to individuals of young age.

In this study, multivariate logistic regression analysis revealed that old age, education attainment, overweight, obese, alcohol use, diabetes, High TC and TG were significantly associated with hypertension. AOR gradually increased with the decrease of education attainment, which is similar with other studies [16], [27]–[29]. Compared with high education attainment, people with low education attainment usually know less knowledge about hypertension and consequently live an unhealthier lifestyle. Numerous studies [3], [6] [16] have found that overweight or obesity was significantly associated with hypertension. Our results were concordant with these studies. It is likely that the increasing prevalence of overweight and obesity in Chinese adults is a major contributing factor to the increased prevalence of hypertension. The present study showed that there was no association between income per capita and hypertension in rural areas, but in urban areas. Lower per capita income, the lower the prevalence of hypertension, which was consistent with Tian *et al.*'s study [30].

The findings of the present study provide important information on factors associated with hypertension awareness, treatment and control in southern China. We found that old age was independently associated with higher hypertension awareness and treatment that are consistent with other reports [9], [31].Our findings show that overweight and obese were associated with better BP awareness but poorer BP control among hypertensive patients. These findings are in agreement with other reports [32], [33], but contrast with that of He *et al.*'s study, in which higher control rates were found among overweight and obese African Americans and White people in the US [34]. It has been suggested that overweight and obesity positively influence blood pressure checking and prescription of medication for intervention; hence, higher awareness and treatment levels [33]. The contrasting results between our study and that of He *et al.*'s study may reflect differences in aggressiveness in treatment of hypertension between these two countries [35].

The associations between education attainment and hypertension awareness and control have remained inconsistent. In Agyemang *et al.*'s study, no associations were found between education level and awareness treatment and control groups of individuals of Indian and African ethnicity, but in the white Dutch hypertensives [32]. In the CHPSNE study [30], lower control rates were found among those with higher education levels. On the contrary, In a multiethnic Asian population's study [36], lower education level was associated with poor BP control, which was consistent with the findings of the present study.

In this study, there were no associations found between awareness and control and cigarette smoking. However, smokers were less likely to receive pharmacological treatment. This implied that physicians should advice smokers with hypertension to quit smoking before they prescribe medication.

Our study has some limitations. First, because this study was a cross-sectional study, the finding cannot be used to establish a conclusive cause-and-effect relationship between risk factors and hypertension. Second, BP was measured two times with only 5 minutes interval. According to the guideline of World Health Organization, hypertension should be defined based on the average of at least two or more BP readings taken at two or more visits after an initial screening. Third, although BP was measured strictly according to guidelines of 1999 WHO-ISH, Manzoli *et al.*'s study showed that the operator's compliance to some recommendations in BP measurement was low [37], which, to some extent, influence the accuracy of the BP measurement.

In conclusion, this study showed an increase in hypertension prevalence among adults in southern China. There was also an increase in hypertension awareness, treatment and control rates. However, hypertension awareness, treatment and control rates were still lower than developed countries. More effort should be targeted to enhance awareness, treatment and control rates, to find hypertensive patients in the early stage, and to strengthen community management of hypertensive patients.

## Supporting Information

Table S1 Percentage (%) of awareness, treatment and control of hypertension among hypertensive adults (N = 5227) in Southern China in 2010.
(DOC)

## References

[1] He J, Gu D, Wu X, Reynolds K, Duan X, et al (2005) Major causes of death among men and women in China. N Engl J Med 353: 1124–1134. 16162883 10.1056/NEJMsa050467

[2] Whitworth JA (2003) 2003 World Health Organization (WHO)/International Society of Hypertension (ISH) statement on management of hypertension. J Hypertens 21: 1983–1992. 14597836 10.1097/00004872-200311000-00002

[3] Wu Y, Huxley R, Li L, Anna V, Xie G, et al (2008) Prevalence, awareness, treatment, and control of hypertension in China: data from the China National Nutrition and Health Survey 2002. Circulation 118: 2679–2686. 19106390 10.1161/CIRCULATIONAHA.108.788166

[4] Zhao L, Stamler J, Yan LL, Zhou B, Wu Y, et al (2004) Blood pressure differences between northern and southern Chinese: role of dietary factors: the International Study on Macronutrients and Blood Pressure. Hypertension 43: 1332–1337. 15117915 10.1161/01.HYP.0000128243.06502.bc PMC6688605

[5] Cai L, Liu A, Zhang L, Li S, Wang P (2012) Prevalence, Awareness, Treatment, and Control of Hypertension among Adults in Beijing, China. Clin Exp Hypertens 34: 45–52. 21967022 10.3109/10641963.2011.618206

[6] Meng XJ, Dong GH, Wang D, Liu MM, Lin Q, et al (2011) Prevalence, awareness, treatment, control, and risk factors associated with hypertension in urban adults from 33 communities of China: the CHPSNE study. J Hypertens 29: 1303–1310. 21558952 10.1097/HJH.0b013e328347f79e

[7] Yang J, Lu F, Zhang C, Liu Z, Zhao Y, et al (2010) Prevalence of prehypertension and hypertension in a Chinese rural area from 1991 to 2007. Hypertens Res 33: 331–337. 20094058 10.1038/hr.2009.235

[8] Liu L, Yu M, Zhong J, Hu R, Chen Y, et al (2007) A Survey on the Nutrition and Health Status of Residents in Zhejiang Province. Zhejiang Prev Med 19: 1–2,82.

[9] Muntner P, Gu D, Wu X, Duan X, Wenqi G, et al (2004) Factors associated with hypertension awareness, treatment, and control in a representative sample of the chinese population. Hypertension 43: 578–585. 14744929 10.1161/01.HYP.0000116302.08484.14

[10] Chalmers J, MacMahon S, Mancia G, Whitworth J, Beilin L, et al (1999) 1999 World Health Organization-International Society of Hypertension Guidelines for the management of hypertension. Guidelines sub-committee of the World Health Organization. Clin Exp Hypertens 21: 1009–1060. 10423121 10.3109/10641969909061028

[11] Kanazawa M, Yoshiike N, Osaka T, Numba Y, Zimmet P, et al (2002) Criteria and classification of obesity in Japan and Asia-Oceania. Asia Pac J Clin Nutr 11 Suppl 8: S732–S737. 10.1159/000088200 16145245

[12] Kearney PM, Whelton M, Reynolds K, Muntner P, Whelton PK, et al (2005) Global burden of hypertension: analysis of worldwide data. Lancet 365: 217–223. 15652604 10.1016/S0140-6736(05)17741-1

[13] Liu Z (2009) Dietary sodium and the incidence of hypertension in the Chinese population: a review of nationwide surveys. Am J Hypertens 22: 929–933. 19661928 10.1038/ajh.2009.134

[14] Ko Ko Z, Tint Swe L, Phyu Phyu A, Thein Gi T, Tin Khine M (2011) Prevalence of hypertension and its associated factors in the adult population in Yangon Division, Myanmar. Asia Pac J Public Health 23: 496–506. 20460273 10.1177/1010539509349147

[15] Joshi V, Lim J, Nandkumar M (2007) Prevalence and risk factors of undetected elevated blood pressure in an elderly Southeast Asian population. Asia Pac J Public Health 19: 3–9. 10.1177/10105395070190020201 18050557

[16] Huang J, Zhang W, Li X, Zhou J, Gao Y, et al (2011) Analysis of the prevalence and risk factors of hypertension in the She population in Fujian, China. Kidney Blood Press Res 34: 69–74. 21212687 10.1159/000323164

[17] Ulasi, II, Ijoma CK, Onwubere BJ, Arodiwe E, Onodugo O, et al (2011) High prevalence and low awareness of hypertension in a market population in enugu, Nigeria. Int J Hypertens 2011: 869675. 21331378 10.4061/2011/869675 PMC3038598

[18] Ashraf MS, Vongpatanasin W (2006) Estrogen and hypertension. Curr Hypertens Rep 8: 368–376. 16965722 10.1007/s11906-006-0080-1

[19] Barengo NC, Kastarinen M, Antikainen R, Nissinen A, Tuomilehto J (2009) The effects of awareness, treatment and control of hypertension on cardiovascular and all-cause mortality in a community-based population. J Hum Hypertens 23: 808–816. 19369956 10.1038/jhh.2009.30

[20] Yoon SS, Ostchega Y, Louis T (2010) Recent trends in the prevalence of high blood pressure and its treatment and control, 1999–2008. NCHS Data Brief 1–8. 21050532

[21] Wyatt SB, Akylbekova EL, Wofford MR, Coady SA, Walker ER, et al (2008) Prevalence, awareness, treatment, and control of hypertension in the Jackson Heart Study. Hypertension 51: 650–656. 18268140 10.1161/HYPERTENSIONAHA.107.100081

[22] Meisinger C, Heier M, Volzke H, Lowel H, Mitusch R, et al (2006) Regional disparities of hypertension prevalence and management within Germany. J Hypertens 24: 293–299. 16508575 10.1097/01.hjh.0000200508.10324.8e

[23] Pereira M, Lunet N, Azevedo A, Barros H (2009) Differences in prevalence, awareness, treatment and control of hypertension between developing and developed countries. J Hypertens 27: 963–975. 19402221 10.1097/hjh.0b013e3283282f65

[24] Vigna-Taglianti F, Vadrucci S, Faggiano F, Burkhart G, Siliquini R, et al (2009) Is universal prevention against youths' substance misuse really universal? Gender-specific effects in the EU-Dap school-based prevention trial. J Epidemiol Community Health 63: 722–728. 19395396 10.1136/jech.2008.081513

[25] Chobanian AV, Bakris GL, Black HR, Cushman WC, Green LA, et al (2003) Seventh report of the Joint National Committee on Prevention, Detection, Evaluation, and Treatment of High Blood Pressure. Hypertension 42: 1206–1252. 14656957 10.1161/01.HYP.0000107251.49515.c2

[26] Williams B, Poulter NR, Brown MJ, Davis M, McInnes GT, et al (2004) Guidelines for management of hypertension: report of the fourth working party of the British Hypertension Society, 2004-BHS IV. J Hum Hypertens 18: 139–185. 14973512 10.1038/sj.jhh.1001683

[27] Erem C, Hacihasanoglu A, Kocak M, Deger O, Topbas M (2009) Prevalence of prehypertension and hypertension and associated risk factors among Turkish adults: Trabzon Hypertension Study. J Public Health (Oxf) 31: 47–58. 18829520 10.1093/pubmed/fdn078

[28] Choi KM, Park HS, Han JH, Lee JS, Lee J, et al (2006) Prevalence of prehypertension and hypertension in a Korean population: Korean National Health and Nutrition Survey 2001. J Hypertens 24: 1515–1521. 16877953 10.1097/01.hjh.0000239286.02389.0f

[29] Prince MJ, Ebrahim S, Acosta D, Ferri CP, Guerra M, et al (2012) Hypertension prevalence, awareness, treatment and control among older people in Latin America, India and China: a 10/66 cross-sectional population-based survey. J Hypertens 30: 177–187. 22134385 10.1097/HJH.0b013e32834d9eda

[30] Tian S, Dong GH, Wang D, Liu MM, Lin Q, et al (2011) Factors associated with prevalence, awareness, treatment and control of hypertension in urban adults from 33 communities in China: the CHPSNE Study. Hypertens Res 34: 1087–1092. 21775998 10.1038/hr.2011.99

[31] Agyemang C, Bruijnzeels MA, Owusu-Dabo E (2006) Factors associated with hypertension awareness, treatment, and control in Ghana, West Africa. J Hum Hypertens 20: 67–71. 16121199 10.1038/sj.jhh.1001923

[32] Agyemang C, van Valkengoed I, Koopmans R, Stronks K (2006) Factors associated with hypertension awareness, treatment and control among ethnic groups in Amsterdam, the Netherlands: the SUNSET study. J Hum Hypertens 20: 874–881. 16929341 10.1038/sj.jhh.1002073

[33] Chen R, Tunstall-Pedoe H, Morrison C, Connaghan J, A'Brook R (2003) Trends and social factors in blood pressure control in Scottish MONICA surveys 1986–1995: the rule of halves revisited. J Hum Hypertens 17: 751–759. 14578914 10.1038/sj.jhh.1001612

[34] He J, Muntner P, Chen J, Roccella EJ, Streiffer RH, et al (2002) Factors associated with hypertension control in the general population of the United States. Arch Intern Med 162: 1051–1058. 11996617 10.1001/archinte.162.9.1051

[35] Agyemang C, Bindraban N, Mairuhu G, Montfrans G, Koopmans R, et al (2005) Prevalence, awareness, treatment, and control of hypertension among Black Surinamese, South Asian Surinamese and White Dutch in Amsterdam, The Netherlands: the SUNSET study. J Hypertens 23: 1971–1977. 16208137 10.1097/01.hjh.0000186835.63996.d4

[36] Wu Y, Tai ES, Heng D, Tan CE, Low LP, et al (2009) Risk factors associated with hypertension awareness, treatment, and control in a multi-ethnic Asian population. J Hypertens 27: 190–197. 19145784 10.1097/hjh.0b013e328317c8c3

[37] Manzoli L, Simonetti V, D'Errico MM, De Vito C, Flacco ME, et al (2012) (In)accuracy of blood pressure measurement in 14 Italian hospitals. J Hypertens 30: 1955–1960. 22902872 10.1097/HJH.0b013e3283577b20

