# Supplementary material for: Factors Associated with Prevalence, Awareness, Treatment and Control of Hypertension among Adults in Southern China: A Community-Based, Cross-Sectional Survey
Source: PLoS One. 2013 May 9;8(5):e62469. doi: 10.1371/journal.pone.0062469 (PMC3650037; doi:10.1371/journal.pone.0062469)
Supplement: Table S1 — Percentage (%) of awareness, treatment and control of hypertension among hypertensive adults (N = 5227) in Southern China in 2010. (DOC) [file pone.0062469.s001.doc]

**Table S1** Percentage (%) of awareness, treatment and control of hypertension among hypertensive adults (N=5227) in Southern China in 2010

|  |  |  |  | Awareness |  |  |  | Treatment |  |  |  | Control |  |
| --- | --- | --- | --- | --- | --- | --- | --- | --- | --- | --- | --- | --- | --- |
|  | N^a^ |  | n^b^(%) | (95%CI) | *P* |  | n^b^(%) | (95%CI) | *P* |  | n^b^(%) | (95%CI) | *P* |
| Total | 5227 |  | 2840(54.33) | 52.98-55.68 |  |  | 2422(46.34) | 44.98-47.69 |  |  | 954(18.26) | 17.20-19.30 |  |
| Men | 2550 |  | 1319(51.73) | 49.78-53.67 | <0.001 |  | 1109(43.49) | 41.56-45.42 | <0.001 |  | 457(17.92) | 16.43-19.41 | >0.05 |
| Women | 2677 |  | 1521(56.82) | 54.94-58.69 |  |  | 1313(49.05) | 47.15-50.94 |  |  | 497(18.57) | 17.09-20.04 |  |
| Urban |  |  |  |  |  |  |  |  |  |  |  |  |  |
| Total | 2386 |  | 1389(58.21) | 56.23-60.19 |  |  | 1223(51.26) | 49.25-53.26 |  |  | 455(19.07) | 17.49-20.65 |  |
| Men | 1160 |  | 653(56.29) | 53.43-59.15 | >0.05 |  | 570(49.14) | 46.26-52.02 | <0.05 |  | 219(18.88) | 16.62-21.13 | >0.05 |
| Women | 1226 |  | 736(60.03) | 57.29-62.78 |  |  | 653(53.26) | 50.47-56.06 |  |  | 236(19.25) | 17.04-21.46 |  |
| Rural |  |  |  |  |  |  |  |  |  |  |  |  |  |
| Total | 2841 |  | 1451(51.07) | 49.23-52.91 |  |  | 1199(42.20) | 40.39-44.02 |  |  | 499(17.56) | 16.16-18.96 |  |
| Men | 1390 |  | 666(47.91) | 45.28-50.54 | <0.01 |  | 539(38.78) | 36.21-41.34 | <0.001 |  | 238(17.12) | 15.14-19.11 | >0.05 |
| Women | 1451 |  | 785(54.10) | 51.53-56.67 |  |  | 660(45.49) | 42.92-48.05 |  |  | 261(17.99) | 16.01-19.97 |  |

^a^Number of hypertensives ^b^Number of hypertensives who were aware of their diagnosis of hypertension ^C^Number of hypertensives receiving antihypertensive medication ^d^Number of hypertensives whose blood level under control.
